# Dietary Intakes of Copper and Selenium in Association with Bone Mineral Density

**DOI:** 10.3390/nu16162777

**Published:** 2024-08-20

**Authors:** Julie A. Pasco, Kara B. Anderson, Lana J. Williams, Amanda L. Stuart, Natalia K. Hyde, Kara L. Holloway-Kew

**Affiliations:** 1 IMPACT-Institute for Mental and Physical Health and Clinical Translation, Barwon Health, Deakin University, Geelong, VIC 3220, Australia; k.anderson@deakin.edu.au (K.B.A.); l.williams@deakin.edu.au (L.J.W.); a.stuart@deakin.edu.au (A.L.S.); natalie.hyde@deakin.edu.au (N.K.H.); k.holloway@deakin.edu.au (K.L.H.-K.); 2 Department of Medicine–Western Health, The University of Melbourne, St Albans, VIC 3021, Australia; 3 Department of Preventive Medicine, Monash University, Melbourne, VIC 3004, Australia

**Keywords:** copper, selenium, trace elements, antioxidant, bone health, Geelong Osteoporosis Study

## Abstract

The important trace elements, copper and selenium, have diverse effects on human health. As well as other important roles in living tissues, these trace elements are toxic at high levels but are key constituents of various enzymes and proteins essential for maintaining physiological health. However, links between dietary intakes of these elements, particularly copper, and bone mineral density (BMD) in humans remain uncertain. This study aimed to investigate whether dietary intakes of copper and selenium are associated with BMD in women. Dietary intakes of copper and selenium were assessed for 575 women in the Geelong Osteoporosis Study, using a detailed semi-quantitative food frequency questionnaire in conjunction with nutrition composition databases. Participants taking oral multivitamin preparations were excluded from analyses; 522 participants (ages 20–88 y) met the eligibility criteria. BMD at multiple skeletal sites was measured by dual energy X-ray absorptiometry (Lunar DPX-L). Separate multivariable regression models were developed to identify associations between copper and selenium intakes and BMD, after adjustments for age, anthropometry, other dietary factors, medication use, and lifestyle factors. Median (interquartile range) daily intake for copper was 1.5 mg (1.2–1.9) and for selenium, 72 μg (57–90). Low intakes (lowest tertile versus pooled upper tertiles) of copper and selenium were consistently associated with lower BMD at multiple skeletal sites. Fully adjusted models identified small but statistically significant differences in BMD, ranging from 1.8% to 4.0% for low copper intakes and 1.4% to 4.0% for low selenium intakes. Low dietary intakes of copper and selenium were both independently associated with lower BMD, at least in this sample of women. The results contribute to the evidence base for informing dietary recommendations for these trace elements with respect to their contributions to optimal bone health.

## 1. Introduction

Copper and selenium are both nutritionally essential trace elements. They are natural constituents of various enzymes with different functions in the human body. The main functions of copper relate to its incorporation into enzymes essential for energy metabolism and in the production of cross-links important in connective tissues, including bone [1,2], where copper functions as a cofactor for lysyl oxidase during the cross-linking processes in collagen fibres [3]. Copper also promotes osteogenesis over adipogenic differentiation of bone mesenchymal stem cells, favouring bone development [4]. Most foods contain some copper, and the quantity of copper absorbed varies with dietary copper intake [5]. While acquired severe copper depletion and toxicity are rare, reports reveal that copper insufficiency is higher than previously realised, causing some impairments to copper-dependent metabolic pathways [6]. Moderate copper insufficiency may predispose individuals at risk of osteoporosis [7,8,9].

Selenium concentrations in foods are largely dependent on the selenium content of soil [10,11]. At high doses, selenium is toxic to humans; however, it is an important component of various enzymes and proteins involved in cell signalling and the maintenance of immunoendocrine function [11]. In the body, selenium is incorporated into selenoproteins, including glutathione peroxidases and thioredoxin reductases; these and other selenoproteins play pivotal roles in maintaining cellular redox balance. Selenoproteins can modulate the NF-κB pathway in macrophages and regulate the expression of a cascade of cytokines that can potentially interfere with the osteoblast stress response and the activation of osteoclasts [12]. While most observational human studies indicate that selenium status correlates positively with bone mineral density (BMD) [13,14], the effects of selenium on bone health remain controversial.

In recognition of the biological activity for these micronutrients, we aimed to investigate whether dietary copper and selenium intakes are associated with BMD in women.

## 2. Materials and Methods

### 2.1. Participants

Participants were enrolled in the Geelong Osteoporosis Study, a population-based study of 1494 women recruited at random from electoral rolls, with 77% participation [15]. The sole inclusion criterion was a listing as a resident of the Barwon Statistical Division in southeastern Australia, but residency in the region for less than six months or inability to provide informed consent necessitated exclusion. This cross-sectional analysis included dietary data for 575 women who completed a food frequency questionnaire and the core study questionnaire, in conjunction with clinical measures, including bone densitometry. After excluding participants with incomplete or anomalous dietary records and those who reported using multivitamin preparations, 522 were eligible for analyses. As there were low numbers of participants using hormone therapy (n = 37) or oral glucocorticoids (n = 3), they were retained in the sample; no participants were taking antifracture medications. The study was approved by the Human Research Ethics Committee at Barwon Health. All participants provided written, informed consent.

### 2.2. Data

Self-reported medication use, diet, fracture history and health behaviours were documented by questionnaire during assessment. Participants were asked to bring in a list of medications or containers to assist with accurate recording of details. Prior fractures resulting from a low-trauma event in adulthood were recorded if they were described as either spontaneous fractures, fractures resulting from strenuous activity, fractures after falls from standing height or less, or “other and unspecified” falls [16]. Participants were classified as alcohol users if they regularly consumed alcohol several times a week or every day. Smoking was classified as current or not. Mobility levels were documented as very active, active, sedentary, limited, inactive, chair/bedridden, or bedfast. Participants were classified as physically active if they were in the very active or active categories; otherwise, they were described as sedentary. Calcium intake was estimated using a calcium-specific questionnaire [17].

A detailed semi-quantitative food frequency questionnaire [18] captured responses on 359 foods. As previously described [19], the questionnaire captures both the frequency and portion size of foods consumed at each meal, together with details about food preparation and cooking methods. The reproducibility of this meal-based questionnaire had been tested previously [18]. Intakes of copper and selenium were individually estimated from contemporary food composition databases, namely the Australia and New Zealand Food Authority (ANZFA; 1996, 1999, 2001) where available, or else, the United States Department of Agriculture (USDA; 1999, 2001, 2002). According to Australian recommendations for women, a low copper intake corresponded to values below the adequate intake of 1.2 mg/day and low selenium, to values below the recommended daily intake of 60 μg/day [20].

Height was measured to the nearest 0.1 cm using a Harpenden stadiometer and weight to the nearest 0.1 kg using electronic scales; body mass index (BMI) was calculated as weight/height^2^ (kg/m^2^). Using a densitometer (Lunar DPX-L, software version 1.31), BMD was measured at the lumbar spine (L2–4; n = 522), femoral neck (n = 520), whole body (n = 520), ultradistal(UD)-forearm (n = 519) and mid-forearm (n = 518). Short-term precision in vivo was 0.6, 1.6, 0.4, 2.1, and 1.1%, respectively. Trained personnel performed clinical assessments.

### 2.3. Statistics

Aggregate descriptive statistics were used to describe participant characteristics. We used histograms to visualise the distribution of variables. Continuous data were summarised by means and standard deviations (SD) or medians and interquartile ranges (IQR), while frequencies and proportions were presented for categorical data. The two-sample t-test was used to compare intergroup differences where normality was observed and a Mann–Whitney U analysis where the distribution of continuous data was skewed. A Chi-square test was used to identify differences between categorical data, employing Fisher’s exact test where any expected cell counts were less than five. 

The distributions of copper and selenium intakes were skewed and grouped into tertiles for consideration in statistical modelling. Analysis of covariance was used in separate regression models setting BMD at each skeletal site as the dependent variable and the tertiles of copper or selenium as the dependent variables. As no intergroup differences in adjusted BMD were observed between the upper two tertiles of copper or selenium, the independent variables were tested in models as the lowest tertile versus the pooled upper tertiles. The lowest tertiles corresponded to daily intakes of copper <1.26 mg and selenium <63 μg. All models included adjustments for age and weight. The age variables were polynomial associations at all skeletal sites except the femoral neck; these polynomial relationships were centred about the mean to avoid collinearity. Backwards elimination was used to identify the best models for predicting age- and weight-adjusted BMD by considering lifestyle factors (smoking, alcohol use, physical activity, energy intake, calcium intake, and use of hormone therapy and oral glucocorticoids). The final models were tested for interaction terms. Analyses were performed using Minitab (v16, Minitab, State College, PA, USA). 

## 3. Results

### 3.1. Dietary Intakes

Median daily intakes were 1.5 mg (IQR 1.2–1.9) for copper and 72 μg (IQR 57–90) for selenium. The main food groups that contributed to the average daily total copper intake were grain products (28%); vegetables (24%); fruit (17%); and all meats or seafood—beef, poultry, lamb, pork, fish, shellfish, and offal (16%). For selenium, the food groups that contributed were wheat products (20%), fish (10%), vegetables (10%), beef (10%), fruit (9%), dairy (9%), and poultry (7%). In total, 124 (55.9%) participants had daily intakes of copper below the adequate intake and 152 (68.5%) had daily intakes of selenium below the recommended daily intake. There were no intakes of these trace elements at toxic levels.

Participant characteristics are presented in Table 1 for those in the lowest tertile versus the pooled upper tertiles for each of copper and selenium intakes. For copper, the median daily intakes were 1.1 mg (IQR 0.9–1.2) for the lowest tertile and 1.7 mg (IQR 1.5–2.0) for the pooled upper tertiles (*p* < 0.001). For selenium, the daily intakes were 51 μg (44–57) and 83 μg (72–101), respectively (*p* < 0.001).

### 3.2. Dietary Intakes and BMD

For raw data, a pattern was observed such that mean BMD at all sites was lower for the lowest tertiles compared to the pooled upper tertiles of dietary intakes of copper and selenium; however, differences were not statistically significant. However, in age- and weight-adjusted models, low copper and selenium intakes were consistently associated with lower mean BMD (Table 2). Further adjustments for other variables identified independent contributions from height at the spine and use of hormone therapy at the UD-forearm for intakes of both copper and selenium. No other variables contributed to the models.

The best models explaining the association between intakes of copper and selenium and BMD included adjustments for age and weight at all BMD sites, plus height for spine-BMD, and use of hormone therapy for UD-forearm. The best models indicated that for low intakes of copper, the mean BMD was 2.7% lower at the lumbar spine (*p* = 0.027), 3.3% lower at the femoral neck (*p* = 0.008), 1.8% lower at the whole body (*p* = 0.003), 4.0% lower at the UD-forearm (*p* = 0.005), and 2.4% lower at the mid-forearm (*p* = 0.006). For intakes of selenium, the patterns were similar. For low selenium intakes, mean BMD was 3.2% lower at the femoral neck (*p* = 0.009), 1.4% lower at the whole body (*p* = 0.024), 4.0% lower at the UD-forearm (*p* = 0.004), 2.6% lower at the mid-forearm (*p* = 0.006), and 2.3% lower at the lumbar spine, although this association was not significant (*p* = 0.067).

## 4. Discussion

In this observational study, we identified that dietary intakes of copper and selenium in the lowest tertiles were individually associated with low BMD at multiple skeletal sites. While differences in BMD were small, ranging from 1.8% to 4.0% for copper intakes and 1.4% to 4.0% for selenium, these associations were independent of age, weight, and use of hormone therapy and were not explained by lifestyle behaviours such as physical activity, smoking, alcohol use, total energy consumption, calcium intakes, or use of oral glucocorticoids. Differences in the spine-BMD were independent of height for low copper intake, but height attenuated the association with regard to low selenium intake. While it is known that low BMD confers an increased risk for fracture, it is also evident that a large fraction of individuals with fragility fractures have normal BMD [21]. In this study, no differences were detected in the proportions of women in the low tertiles of copper or selenium versus the upper pooled tertiles of each element.

Plasma levels of copper have been correlated with BMD at the lumbar spine in women, suggesting a role for copper in bone health [22]. Data from 722 participants from the National Health and Nutrition Examination Survey (NHANES) in the USA indicated that people with low serum concentrations of copper had lower mean BMD at the total femur and femoral neck; in contrast, high levels of serum copper were associated with increased fracture risk [7]. However, few human studies in the extant literature have investigated copper intakes and BMD. In a study of postmenopausal women referred to a rheumatology clinic in Iran, mean dietary intake of copper appeared lower for women with osteoporosis compared to those with osteopenia; however, the intergroup difference was not significant [23]. It should be noted that in this study, there were small numbers of participants in each group (n = 28 with osteopenia and n = 23 with osteoporosis) and no adjustments had been made for potential confounders. In a study of men in the UK, which investigated the effects on markers of bone metabolism in response to altering dietary copper intakes, urinary markers of bone resorption were found to increase when copper intakes changed from medium to low levels, and subsequently decreased when the diet was changed from low to high levels of copper [8]. While this study was conducted in men, the results somewhat align with findings from our study in women. There is also some evidence that dietary copper supplementation over two years reduced the loss in the vertebral trabecular BMD in middle-aged women [24].

Recognised as a dietary antioxidant, selenium deficiency has been associated with an elevated risk of several metabolic disturbances including poor glycaemic control, unhealthy lipid profiles such as those associated with obesity and ageing, and thyroid hormone dysfunction [25], while moderate to high levels are protective against cardiovascular disease [26] and mood disturbances [19]. A recent review that systematically evaluated observational cross-sectional data from eight studies from the literature also described associations between dietary selenium intake and BMD, in which the overall association was positive [13]. Results from individual studies, however, were rather mixed. Data from several waves of the NHANES study in the USA that reported a positive association between dietary selenium and DXA-measured BMD also identified a nonlinear inverted U-shape association [27]. The authors noted that increases in dietary selenium were positively associated with BMD when the selenium intakes were low, and that this association either plateaued or became negative for higher intakes of selenium. These findings are similar to the ones reported in our study. A study of postmenopausal women from the Women’s Health Initiative (WHI) indicated a positive association between dietary selenium intake and BMD of the femoral neck (and other sites) after adjusting for age; however, the association was not sustained after accounting for other factors known to affect BMD [28]. Another cross-sectional analysis involving data from the NHANES, this time focusing on elderly individuals, identified positive relationships of intakes of a cocktail of dietary antioxidants with multisite measures of BMD, and a consistent pattern was observed when dietary selenium was considered independently of the other antioxidants [29].

Our study has several strengths and weaknesses. A major strength is the randomly selected nature of the study sample, and a notable characteristic is that participants were not selected on the basis of disease and were representative of the underlying population from which they were drawn. We acknowledge the limitation of relying on self-reported dietary intakes that were documented using a food frequency questionnaire. As with all similar epidemiological studies, there are possible recall biases and inaccuracies. Furthermore, by using food composition databases that assume a standard nutrient content in foods, we are unlikely to have accounted for individual variations in food sources, and this may have led to discrepancies between estimated and actual nutrient intakes. However, dietary data were collected and analysed independently of BMD measurements. In developing statistical models, we adjusted for several potential confounders, including age. To account for non-linear relationships between age and BMD, best-fit models considered age as a polynomial variable. However, we acknowledge that these adjustments might not have adequately addressed intergroup age differentials in dietary intakes or accounted for possible confounding by other unmeasured factors which could have influenced the observed associations between trace element intakes and BMD. Given that our data were pertinent for a population of predominantly white women living in south-eastern Australia, results might not apply to men or women from different ethnic or geographical backgrounds. Similar data are being collected for men enrolled in the Geelong Osteoporosis Study.

## 5. Conclusions

In this observational study, low dietary intakes of copper and selenium were associated with low BMD at multiple sites. The results add to the evidence base for informing dietary recommendations for these trace elements with respect to their contributions to optimal bone health in women.

## Figures and Tables

**Table 1:** Participant characteristics according to copper and selenium intakes, lowest tertiles versus upper tertiles combined. Data are shown as mean (±SD), median (IQR), or n (%).

	Copper			Selenium		
	Lowest Tertile	Upper Tertiles	*p*	Lowest Tertile	Upper Tertiles	*p*
	n = 174	n = 348		n = 175	n = 347	
Age (y)	46.0 (32.5–64.1)	52.8 (37.0–66.0)	0.024	46.6 (34.1–63.3)	52.3 (36.4–65.8)	0.082
Weight (kg)	68.4 (±14.1)	68.2 (±12.2)	0.859	68.5 (±13.7)	68.1 (±12.4)	0.729
Height (cm)	161.8 (±6.6)	161.2 (±6.2)	0.280	161.4 (±6.5)	161.4 (±6.2)	0.958
BMI (kg/m^2^)	26.1 (±5.0)	26.3 (±4.8)	0.658	26.3 (±5.0)	26.2 (±4.8)	0.803
BMD * (g/cm^2^)						
spine	1.166 (±0.184)	1.183 (±0.181)	0.322	1.168 (±0.183)	1.181 (±0.182)	0.440
femoral neck	0.918 (±0.165)	0.931 (±0.153)	0.392	0.917 (±0.162)	0.932 (±0.154)	0.317
whole body	1.126 (±0.107)	1.136 (±0.108)	0.314	1.130 (±0.107)	1.134 (±0.108)	0.678
UD-forearm	0.305 (±0.055)	0.313 (±0.064)	0.129	0.306 (±0.057)	0.313 (±0.063)	0.209
mid-forearm	0.665 (±0.088)	0.671 (±0.091)	0.416	0.665 (±0.089)	0.671 (±0.090)	0.452
Prior fracture	31 (17.8%)	47 (13.5%)	0.193	26 (14.9%)	52 (15.0%)	0.969
Hormone therapy	12 (6.9%)	25 (7.2%)	0.904	16 (9.1%)	21 (6.1%)	0.194
Oral glucocorticoids	1 (0.6%)	2 (0.6%)	1	1 (0.6%)	1 (0.6%)	1
Physically active	129 (74.1%)	262 (75.3%)	0.775	133 (76.0%)	258 (74.4%)	0.682
Smoker	25 (14.4%)	36 (10.3%)	0.177	19 (10.9%)	42 (12.1%)	0.676
Alcohol	36 (20.7%)	70 (20.1%)	0.878	35 (20.0%)	71 (20.5%)	0.902
Dietary intakes						
energy (MJ)	6.1 (5.2–7.3)	8.8 (7.6–10.7)	<0.001	6.1 (5.2–7.2)	8.8 (7.7–10.8)	<0.001
calcium (mg)	557 (365–767)	632 (428–889)	0.006	492 (308–714)	677 (460–906)	<0.001

Abbreviations: BMI, body mass index; BMD, bone mineral density; UD-forearm, ultradistal-forearm; * Missing data: BMD femoral neck n = 2, whole body n = 2, UD-forearm n = 3, mid-forearm n = 4.

**Table 2:** Linear regression models for the lowest tertile versus the pooled upper tertiles for (a) copper and (b) selenium intakes in association with bone mineral density at multiple skeletal sites.

	Variable	Beta Coef	SE Coef	*p*
(a) Copper				
BMD spine	Lowest tertile	−0.03196	0.01468	0.030
	(age-mean) *	−0.0079237	0.0008789	<0.001
	(age-mean)^2^	−0.00010037	0.00002526	<0.001
	(age-mean)^3^	0.00000685	0.00000138	<0.001
	weight	0.0035211	0.0005465	<0.001
BMD femoral neck	Lowest tertile	−0.03093	0.01158	0.008
	(age-mean)	−0.0043565	0.0003131	<0.001
	(age-mean)^2^	-	-	-
	(age-mean)^3^	-	-	-
	weight	0.0047170	0.0004257	<0.001
BMD whole body	Lowest tertile	−0.021007	0.006990	0.003
	(age-mean)	−0.0039753	0.0004184	<0.001
	(age-mean)^2^	−0.00008278	0.00001203	<0.001
	(age-mean)^3^	0.00000198	0.00000066	0.003
	weight	0.0039798	0.0002601	<0.001
BMD UD-forearm	Lowest tertile	−0.013200	0.004740	0.006
	(age-mean)	−0.0019645	0.0002840	<0.001
	(age-mean)^2^	−0.00006452	0.00000814	<0.001
	(age-mean)^3^	0.00000098	0.00000044	0.029
	weight	0.0008687	0.0001762	<0.001
BMD mid-forearm	Lowest tertile	−0.016453	0.005961	0.006
	(age-mean)	−0.0038395	0.0003569	<0.001
	(age-mean)^2^	−0.00011123	0.00001025	<0.001
	(age-mean)^3^	0.00000207	0.00000056	<0.001
	weight	0.0017776	0.0002215	<0.001
(b) Selenium				
BMD spine	Lowest tertile	−0.02820	0.01464	0.055
	(age-mean)	−0.0079088	0.0008797	<0.001
	(age-mean)^2^	−0.00010295	0.00002528	<0.001
	(age-mean)^3^	0.00000688	0.00000138	<0.001
	weight	0.0035218	0.0005470	<0.001
BMD femoral neck	Lowest tertile	−0.03027	0.01155	0.009
	(age-mean)	−0.0043367	0.0003125	<0.001
	(age-mean)^2^	-	-	-
	(age-mean)^3^	-	-	-
	weight	0.0047253	0.0004259	<0.001
BMD whole body	Lowest tertile	−0.015833	0.006992	0.024
	(age-mean)	−0.0039564	0.0004199	<0.001
	(age-mean)^2^	−0.00008437	0.00001208	<0.001
	(age-mean)^3^	0.00000199	0.00000066	0.003
	weight	0.0039784	0.0002611	<0.001
BMD UD-forearm	Lowest tertile	−0.012806	0.004734	0.007
	(age-mean)	−0.0019651	0.0002842	<0.001
	(age-mean)^2^	−0.00006563	0.00000814	<0.001
	(age-mean)^3^	0.00000099	0.00000045	0.026
	weight	0.0008672	0.0001763	<0.001
BMD mid-forearm	Lowest tertile	−0.017365	0.005946	0.004
	(age-mean)	−0.0038447	0.0003567	<0.001
	(age-mean)^2^	−0.00011268	0.00001024	<0.001
	(age-mean)^3^	0.00000210	0.00000056	<0.001
	weight	0.0017764	0.0002213	<0.001

Abbreviations: BMD, bone mineral density; UD-forearm, ultradistal-forearm; * mean = 50 y.

## Data Availability

The data that support the findings of this study are available from the corresponding author upon reasonable request.
